# Fistule artério-veineuse ulno-basilique pour hémodialyse au CHU de Yaoundé: à propos de deux cas

**DOI:** 10.11604/pamj.2013.15.103.2906

**Published:** 2013-07-18

**Authors:** Marc Leroy Guifo, Francois Folefak Kaze, Aurélien Ndoumbe, Marie Patrice Halle, Louis Joss Bitang, Christopher Tagnyin Pisoh, Samuel Takongmo

**Affiliations:** 1 Faculté de Médecine et des Sciences Biomédicales Université de Yaoundé 1, Cameroun; 2 Faculté de Médecine et des Sciences Pharmaceutiques. Université de Douala, Cameroun; 3 Faculté de Médecine CHU de Yaoundé, Cameroun; 4 Université de Yaoundé 1, Cameroun

**Keywords:** Fistule artério-veineuse, hémodialyse, ponction veineuse

## Abstract

La fistule ulno-basilique est une éventualité peu sollicitée dans le choix des abords vasculaires pour hémodialyse. Elle est construite sur la veine basilique qui est souvent la seule veine épargnée par les ponctions veineuses de routine dans les services de médecine. De par l'existence de nombreuses complications comme l'infection des prothèses et des cathéters centraux, les thromboses, les patients hémodialysés chroniques nécessitent plus d'un accès veineux au cours de leur suivi médical. Les fistules artério-veineuses natives présentent moins de risques de complications que les prothèses d'une façon générale et une plus longue durée de vie. La confection d'une fistule sur une topographie proximale compromet le recourt à cette veine sur une localisation distale ultérieurement. Il existe donc une hiérarchisation dont il faut tenir compte pour une utilisation optimale du capital veineux disponible. Nous rapportons ici deux observations concernant des patients chez qui deux fistules ulno-basiliques ont été réalisées avec succès au CHU de Yaoundé.

## Introduction

L'hémodialyse est l'une des modalités de traitement de suppléance de l'insuffisance rénale chronique. Elle nécessite un accès vasculaire capable de délivrer un débit sanguin entre 200 et 600 ml /min pour permettre une filtration sanguine adéquate [1]. La fistule artério-veineuse native reste l'accès vasculaire de choix devant les prothèses et les cathéters veineux centraux en raison de sa longévité et du faible risque de complications infectieuses et thrombotiques [1, 2]. Bien que de nombreux sites soient possibles pour la confection de ces fistules ainsi que la possibilité de recourir à des prothèses vasculaires ou la pose de cathéters, on peut arriver à un épuisement des possibilités si une utilisation judicieuse n'est pas planifiée. Il est recommandé de recourir à une fistule artério-veineuse native le plus souvent car elles ont une plus grande longévité. Les patients nécessitant une hémodialyse au long court ont vu leur espérance de vie augmenter par les progrès de la technologie médicale et doivent utiliser plus d'une fistule pour la plupart au court de leur suivi [3]. La veine basilique localisée à la face interne du membre supérieur est souvent épargnée par les ponctions veineuses de routine dans les services médicaux ; elle offre de nombreuses alternatives à la réalisation d'abord vasculaire pour la dialyse soit sous la forme d'une fistule ulno-basilique directe ou après transposition à l'avant-bras ou au bras. Nous rapportons ici le cas pour deux patients qui présentaient à l'examen initial un capital veineux réduit dans le but de contribuer à une meilleure connaissance de cette alternative [4].

## Patient et observation

### Cas 1

**Figure 1:**
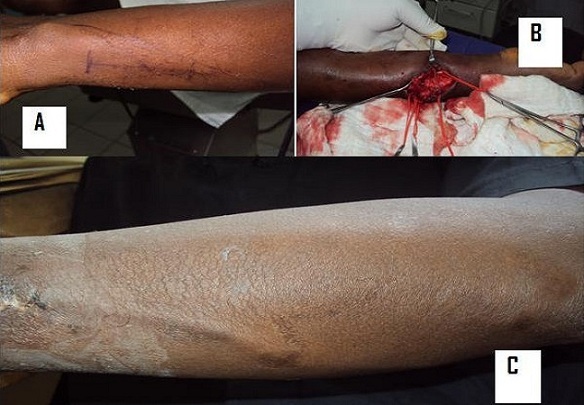
A) marquage préopératoire des trajets vasculaire; à noter divergence distale veine et artère ulnaire; B) image per opératoire de l'opération, 1C: résultat à 3 semaines post opératoire; C) résultat à 3 semaines post opératoire

Mr T D âgé de 53 ans employé des télécommunications et marié est adressé en chirurgie pour la réalisation d'un abord vasculaire pour hémodialyse. Il est suivi en néphrologie pour une insuffisance rénale chronique terminale secondaire à une néphropathie diabétique. Il présentait également une hypertension artérielle et une hépatite virale C chronique non active. A sa consultation initiale on notait que les veines céphaliques sur le membre gauche et droit étaient inapparentes même après la pose d'un garrot. L'hypertension artérielle était contrôlée par une quadrithérapie ainsi que le diabète avec une hémoglobine glyquée à 7%. Un bilan biologique fait montrait une urée à 2,45 g/l, une créatinémie à 102 mg/l. Il présentait par ailleurs une anémie à 9,2 g/dl normocytaire normochrome et sa coagulation était normale. On va lui confectionner le 8 novembre 2012 une fistule ulno-basilique (Figure 1). Devant une l'altération de l'état clinique, il est admis en dialyse le 24 novembre 2012 et il est dialysé par cathéter veineux central qui sera enlevé le 21 décembre en raison d'une infection. Dès lors la dialyse sera effectuée par sa fistule c′est-à-dire 6 semaines après sa réalisation jusqu'au décès survenu le 18/03/2013 de suite d'un infarctus du myocarde.

### Cas 2

**Figure 2:**
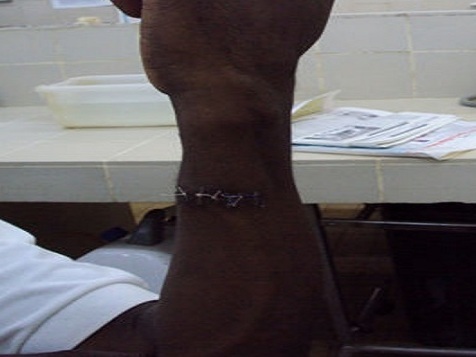
Fistule ulno-basilique. NB compression au doigt au coude par le patient le patient

Mr N M âgé de 63 ans est adressé en chirurgie pour la réalisation d'un abord vasculaire pour hémodialyse. Il est en hémodialyse chronique par cathéter jugulaire interne droit depuis le premier octobre 2012 pour une insuffisance rénale chronique terminale secondaire à une néphropathie diabétique et hypertensive. On lui réalise une FAV radio-céphalique le 18/10/2012 qui sera utilisé le 6/12/2012 soit 6 semaines après; dans son évolution il va présenter une infection du gros orteil gauche et sera à nouveau adressé en chirurgie pour prise en charge. A son admission le 3/01/2013 il présente une infection évolutive de stade 4 de la classification de Wagner, le pouls pédieux est perceptible et la sensibilité tactile est conservée. On lui propose la réalisation sans délai d'une amputation de l'avant pied mais le patients oppose un refus; devant l'extension de l'infection, il va donner son consentement à la réalisation d'une amputation de type syme le 21/01/2013. L'évolution sera marquée par une nécrose du lambeau plantaire après sa sortie du service et une deuxième amputation sera réalisée le 26/03/2013 au tiers supérieur de la jambe d'évolution favorable. Durant son séjour il sera noté une perte de la perméabilité de sa fistule nécessitant la remise d'un cathéter central et la réalisation le 13/02/2013 d'une autre fistule ulno-basilique sur le même membre pour préserver toutes les chances compte tenu des aléas possibles au cours de l'utilisation des fistules (Figure 2).

## Discussion

L'abord vasculaire permanent pour dialyse constitue un élément fondamental pour réaliser une hémodialyse adéquate. Leur fonctionnement optimal et leur longévité impactent la qualité des soins ainsi que l'espérance de vie. Ce fonctionnement est conditionné par le choix du site, le caractère natif et la technique chirurgicale.

Les veines du membre supérieur sont souvent sollicitées pour les prélèvements et les diverses voies de perfusion au cours des traitements; Lors de l'examen pré-opératoire pour la planification d'une fistule, un examen du réseau veineux superficiel et des artères s'impose pour se figurer l'architecture de l'anastomose à réaliser; Du fait de sa situation à la face médiale du membre supérieur, la veine basilique est souvent épargnée lors du choix des sites de ponction et peut être la seule disponible pour la réalisation d'une fistule native ceci était le cas de notre patient TD. Elle diverge au tiers inférieur de l'avant-bras de l'artère ulnaire ce qui impose une légère translation pour parvenir à l'artère sans kinking susceptible de compromettre la perméabilité ou le débit de la fistule. Il est recommandé de recourir d'abord à des fistules natives avant de recourir le cas échéant à des prothèses pour la réalisation de ces abords [1–3].

Le fonctionnement des fistules par ailleurs dépend de la technique chirurgicale. Nous réalisons cette anastomose selon la description technique publiée par Tadahiko TOKUMOTO [5]. Le patient est examiné préalablement pour identifier sous garrot la veine et apprécier son calibre. Les pouls distaux au membre supérieur à savoir radial et ulnaire sont vérifiés. On effectue un marquage au marqueur indélébile pour permettre d'approcher les vaisseaux sans les léser; nous procédons à l'anesthésie locale à la lidocaine non adrénalinée. Nous n'utilisons pas de lunette grossissante ou de microscope opératoire car cette instrumentation n'est pas disponible. En per opératoire la perméabilité de la veine est vérifiée en la purgeant avec du sérum salé à 0.9% héparine à raison de 10000 UI pour 500cc. Après la purge la veine est dilatée par une injection sous pression permettant d'augmenter de 2à 3 fois le diamètre du segment veineux à anastomoser sur l'artère. L'anastomose est réalisée sur une longueur de 0.8 à 1 cm le plus souvent latéro-latérale par un surjet au monofil 7/0 non résorbable. Nous apportons un artifice à notre connaissance non décrit en utilisant si nécessaire le moignon veineux restant comme moyen d'orientation et d'ancrage de l'anastomose en cas de nécessité. Ceci permet de favoriser des conditions hémodynamiques optimales dans l'anastomose et éviter un kinking des vaisseaux qui peuvent affaiblir le débit sanguin dans la fistule [6].

Les fistules artério-veineuses doivent atteindre une certaine maturité au bout de quatre à six semaines avant le début du piquage en dialyse. Ce délai permet l'augmentation du diamètre, une exposition suffisante pour une ponction sécurisée et finalement un accroissement du débit dans la fistule qui doit atteindre au moins 600ml/min.

Bien que peu de publications soient faites sur les fistules artério-veineuses ulno-basiliques, elles ont la réputation de nécessiter plus de temps pour atteindre la maturité en comparaison aux fistules radio-céphaliques. Il est donc souhaitable de les confectionner suffisamment à l'avance pour permettre leur maturation avant le recourt à la dialyse. Dans les recommandations Nord-américaines en général les patients doivent être adressés pour fistules 3 à 6 mois avant le déclin critique de la filtration glomérulaire en dessous de 0ml/min [1]. Chez nos patients ceci n'avait pas été le cas et une fistule a dû être utilisé précocement à 6 semaines en raison des signes infectieux présenté par le cathéter. L'Evaluation Doppler du débit des fistules avant piquage n'a pas été effectuée chez nos patients en raison des contraintes locales. Toutes les deux fistules ont été utilisées pour dialyse pendant 4 mois pour le patient TD et jusqu'à ce jour pour le patient NM (2 mois).

Les fistules artério-veineuses natives sont aussi préférées aux prothèses en raison d'un risque infectieux moindre. Cette considération est encore plus importante lorsqu' on se trouve dans notre environnement tropical oé les risques d'infections nosocomiales sont plus importants notamment après la mise en place d'un matériel prothétique.

Nous n'avons pas eu pour ces deux patients une plainte évoquant une ischémie de la main. On sait que certaines fistules du fait d'un débit très élevé peuvent être délétères pour la perfusion distale dans 28% pour les abords proximaux à l'avant-bras et peuvent engendrer une insuffisance cardiaque [7, 8].

## Conclusion

Il existe une hiérarchisation des différents accès pour dialyse qui ne prennent pas en compte la fistule ulno-basilique probablement pour son manque de popularité et les réserves que nous avons relevés. A notre avis la fistule ulno-basilique viendrait en seconde considération après les fistules radio-céphalique distale et proximale à l'avant-bras car les autres possibilités plus proximales brachio-brachiale avec ou sans transposition éliminent de fait cette alternative. Ce site augmenterait les choix possibles sans compromettre les possibilités en amont d'utilisation de la même veine.
